# The efficacy and safety of hydroxychloroquine versus leflunomide in patients with IgA nephropathy: a single-center experience

**DOI:** 10.1007/s40620-023-01839-x

**Published:** 2024-01-16

**Authors:** Wei-jie He, Juan Wang, Nan Liu, Gu-yue Li, Xin-wang Zhu, Li Yao, Lin-lin Liu

**Affiliations:** 1 Department of Nephrology, The First Affiliated Hospital of China Medical University, 155 Nan Jing North Street, He Ping District, Shenyang, 110001 Liaoning China; 2 Department of Pharmacy, The First Hospital of China Medical University, Shenyang, 110001 China

**Keywords:** IgA nephropathy, Hydroxychloroquine, Leflunomide, Proteinuria, Hematuria

## Abstract

**Purpose:**

To date, our understanding of IgA nephropathy (IgAN) pathophysiology has remained incomplete; therefore, treatment remains largely empiric, and the efficacy and safety of immunosuppressants remain controversial. We aimed to assess the efficacy and safety of hydroxychloroquine and leflunomide therapy in a retrospective cohort of patients with IgAN.

**Methods:**

We screened the IgAN registration database in our department, and a total of 159 kidney patients with biopsy-confirmed IgAN were enrolled, with 57 patients receiving hydroxychloroquine plus a renin-angiotensin system inhibitor (hydroxychloroquine group), 52 patients receiving leflunomide plus a renin-angiotensin system inhibitor (leflunomide group), and 50 patients receiving only a renin-angiotensin system inhibitor (renin-angiotensin system inhibitor-only group). Changes in proteinuria, hematuria, and the estimated glomerular filtration rate (eGFR), as well as adverse events, were analyzed during the follow-up period.

**Results:**

At the end of 6-month follow-up, proteinuria significantly decreased by 70.36 (57.54, 79.33)%, 57.29 (46.79, 67.29)% and 41.20 (25.76, 48.94)% in the hydroxychloroquine, leflunomide and renin-angiotensin system inhibitor-only groups, respectively, compared to baseline (all *P* values < 0.001). Hematuria significantly decreased by 71.07 (56.48, 82.47)% in the leflunomide group (*P* < 0.001). The eGFR improved by 3.72 ± 2.97%, 3.16 ± 2.00% and 1.91 ± 2.41%, respectively, in the hydroxychloroquine, leflunomide and renin-angiotensin system inhibitor-only groups, but without statistical significance. No serious adverse events occurred during the follow-up period.

**Conclusion:**

Both hydroxychloroquine combined with a renin-angiotensin system inhibitor and leflunomide combined with a renin-angiotensin system inhibitor were more effective than a renin-angiotensin system inhibitor alone in improving proteinuria in IgAN patients. Hydroxychloroquine was more effective in reducing proteinuria, and leflunomide showed superiority in reducing hematuria. Our results need to be verified in large-scale randomized controlled trials.

**Graphical abstract:**

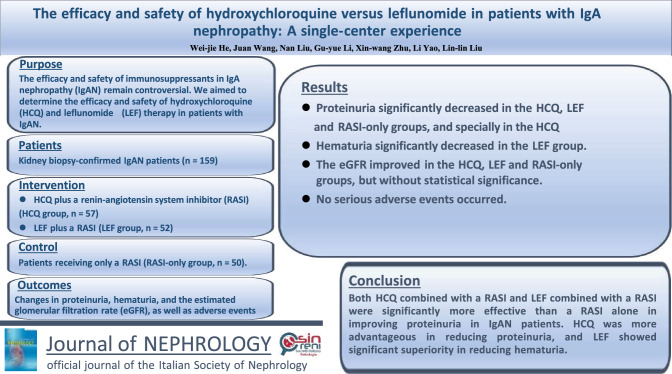

## Introduction

IgA nephropathy (IgAN) is one of the most common primary glomerulonephritides, especially in Asia, and it is a leading cause of end-stage renal disease [1–3]. The clinical and pathological manifestations of IgAN are variable, and therefore, the prognosis is diverse. According to previous studies, many factors can aid in predicting the prognosis of IgAN, such as hematuria [4], proteinuria, hypertension, renal function and pathological factors [5–7]. To date, the pathophysiological mechanism has remained unclear, and treatment remains largely empiric. In addition, the safety and efficacy of steroids and immunosuppressants remain controversial [8–10]. Various immunosuppressive agents may be effective in subgroups of IgAN patients based on various mechanisms of action.

Hydroxychloroquine, a well-known antimalarial and immunomodulatory agent, has been used in various autoimmune diseases [11]. It significantly affects immune activation by reducing circulating activated immune cells, including TLR-expressing cells and IFN-secreting dendritic cells, and the production of cytokines such as IFNα, IL-6 and TNF-α [12, 13]. In recent years, some studies have reported that hydroxychloroquine may reduce the production of pathogenic IgAs [14]. In addition, a few clinical trials also found that hydroxychloroquine can reduce proteinuria in IgAN patients [15–17].

Leflunomide is an immunosuppressive agent that is widely used to treat rheumatoid arthritis, systemic lupus erythematosus and organ transplant rejection. Leflunomide inhibits lymphocyte dihydroorotate dehydrogenase to produce immunosuppressive effects and blocks tyrosine kinase activation to generate anti-inflammatory effects [18, 19]. Currently, there are not enough clinical studies on leflunomide for IgAN, and due to limited sample sizes, these studies have come to some conflicting conclusions [20, 21].

The present study was performed to retrospectively evaluate the efficacy and safety of hydroxychloroquine and leflunomide in patients with IgAN with moderate proteinuria.

## Materials and methods

### Study population

The present study is a single-center, retrospective analysis. We screened the IgAN registration database in our department and included patients according to the following criteria: (1) adult patients who underwent renal biopsy and were confirmed to have primary IgAN from January 2018 to December 2021; (2) in the immunosuppressive groups, patients who were treated with hydroxychloroquine or leflunomide as the only immunosuppressant, and in the control group, sex- and age-matched patients who were treated only with renin-angiotensin system inhibitors; and (3) patients in the immunosuppressive groups who had been treated with hydroxychloroquine or leflunomide for at least 6 months. The exclusion criteria included secondary IgAN (e.g., due to Henoch–Schönlein purpura, systemic lupus erythematosus, liver disease, ankylosing spondylitis, psoriasis, etc.), a lack of baseline or follow-up data, pregnancy and treatment with steroids or other immunosuppressants within 3 months before or during hydroxychloroquine or leflunomide therapy. Our study was conducted according to the principles of the Declaration of Helsinki and was approved by the Institutional Animal Care and Use Committee (IACUC) of China Medical University.

### Treatments

The oral dosage of hydroxychloroquine was 0.2 g orally twice daily for patients with an eGFR > 60 ml/min/1.73 m^2^, 0.1 g orally 3 times daily for patients with an eGFR between 45 and 59 ml/min/1.73 m^2^, and 0.1 g orally twice daily for patients with an eGFR between 30 and 44 ml/min/1.73 m^2^ [22]. The oral dosage of leflunomide was 20 mg orally once daily [23, 24]. Renin-angiotensin system inhibitors were administered at the maximum tolerated dose in both the immunosuppressive groups and the control group.

### Data collection and outcome measures

The following characteristics were collected before the renal biopsy as the baseline data: age, sex, renal pathology according to the Oxford Classification, the proportion of patients with hypertension, 24-h total urinary protein excretion, urinary red blood cell counts per high power field (u-RBC/HPF), serum creatinine level and eGFR. The eGFR was calculated with the Chronic Kidney Disease Epidemiology Collaboration (CKD-EPI) formula using serum creatinine. The following indicators were collected at months 1, 3 and 6 after the patient received treatment: 24-h total urinary protein excretion, urinary red blood cell counts per high power field, eGFR, therapeutic regimens, and adverse events.

Medical events that met one or more of the following criteria were defined as serious adverse events: death, life-threatening, required inpatient hospitalization or the prolongation of an existing hospitalization, resulted in persistent or significant disability, involved a severe infection that required hospitalization, new-onset retinopathy or visual field impairment, severe liver dysfunction or allergies that required hospitalization, and major cardiocerebral events. Adverse events were collected from the patient’s medical records.

### Statistical analysis

SPSS 23.0 software was used for data analysis. Normally distributed variables are expressed as the mean ± standard deviation (SD) and compared using an independent or paired *t* test, as appropriate. Nonparametric continuous variables are presented as median and interquartile range (IQR, 25th and 75th percentile), and nonparametric tests were used for comparison when appropriate. The *χ*^2^ test was used to compare categorical data. A *P* value of < 0.05 was considered statistically significant.

## Results

### Baseline characteristics of the included patients

Fifty-seven patients were treated with hydroxychloroquine combined with a renin-angiotensin system inhibitor, and 52 patients were treated with leflunomide combined with a renin-angiotensin system inhibitor. We chose 50 sex- and age-matched patients who were treated with a renin-angiotensin system inhibitor alone. The baseline characteristics of the patients in the three groups are shown in Table 1. There were no statistically significant differences in the age, sex ratio, proportion of patients with hypertension, eGFR, serum albumin, serum alanine aminotransferase, 24-h total urinary protein excretion or pathological classification among the three groups. However, hematuria in the leflunomide group was more serious than that in the other two groups (*P* < 0.001).

**Table 1:** Baseline characteristics of the leflunomide group, HCQ group and RASI-only group

	HCQ group (*n* = 57)	LEF group (*n* = 52)	RASI only group (*n* = 50)	*P* value
Age (years)	39.26 ± 11.84	39.25 ± 11.51	39.48 ± 12.07	0.994
Male (%)	52.63	48.08	56	0.723
Hypertension (%)	31.58	26.92	26	0.786
eGFR (ml/min/1.73 m^2^)	74.83 ± 14.14	79.86 ± 10.19	79.26 ± 17.22	0.128
Serum albumin (g/L)	39.13 ± 4.24	40.86 ± 2.88	39.79 ± 6.89	0.167
Proteinuria (g/d)	0.89 (0.70, 1.23)	0.84 (0.71, 1.07)	0.77 (0.67, 0.96)	0.138
Hematuria (u-RBC/HPF)	11.62 (4.09, 24.20)	43.75 (31.33, 67.90)	14.17 (3.40, 19.73)	< 0.001
ALT(U/L) Oxford classification	25 (22, 27)	25 (21, 30)	25.5 (22, 29.25)	0.873
M (M0/M1)	24/33	29/23	22/28	0.312
E (E0/E1)	38/19	42/10	38/12	0.229
S (S0/S1)	10/47	12/40	9/41	0.728
T (T0/T1/T2)	40/14/3	45/4/3	38/10/2	0.218
C (C0/C1/C2)	55/2/0	47/5/0	47/3/0	**–**

*HCQ* Hydroxychloroquine, *LEF* leflunomide*, RASI *renin-angiotensin system inhibitor, *eGFR* estimated glomerular filtration rate, *RBC* red blood cell, *HPF* high power field, *ALT* alanine aminotransferase

### Proteinuria

There were no significant differences in urinary protein level among the three groups at baseline (*P* = 0.138). After 6 months of treatment, urinary protein levels decreased significantly in all three groups [hydroxychloroquine group: 0.89 (0.70, 1.23) to 0.22 (0.14, 0.49) g/d, *P* < 0.001; leflunomide group: 0.84 (0.70, 1.07) to 0.37 (0.22,0.64) g/d, *P* < 0.001; renin-angiotensin system inhibitor-only group: 0.77 (0.67, 0.96) to 0.47 (0.36, 0.69) g/d, *P* < 0.001] (Table 2).

**Table 2:** Changes in 24-h total urine protein (g/d)

	Month0	Month1	Month3	Month6	*P-*value
HCQ group	0.89 (0.70, 1.23)	0.72 (0.51, 1.00)	0.52 (0.38, 0.75)	0.22 (0.14, 0.49)	< 0.001
LEF group	0.84 (0.70, 1.07)	0.74 (0.59, 0.98)	0.56 (0.44, 0.81)	0.37 (0.22, 0.64)	< 0.001
RASI-only group	0.77 (0.67, 0.96)	0.64 (0.55, 0.88)	0.53 (0.41, 0.71)	0.47 (0.36, 0.69)	< 0.001
*P*-value	0.138	0.481	0.395	< 0.001	–

*HCQ* Hydroxychloroquine, *LEF* leflunomide*, RASI *renin-angiotensin system inhibitor

**Fig. 1:**
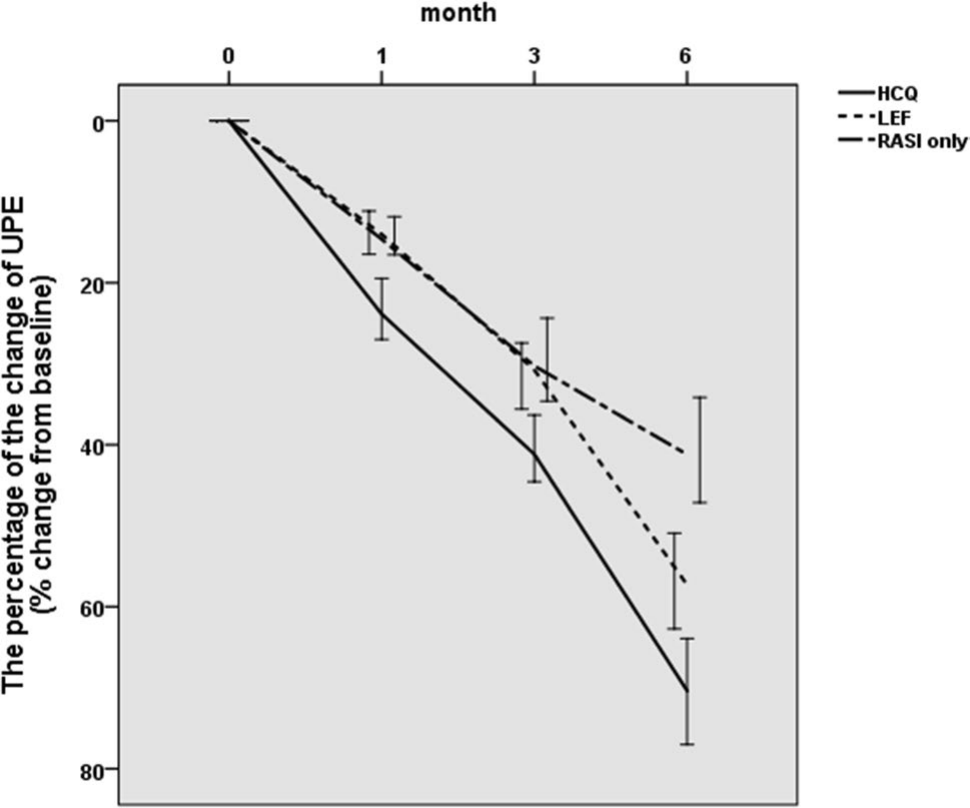
The percentage change in UPE as a function of treatment. *HCQ* hydroxychloroquine; *LEF* leflunomide; *RASI* renin-angiotensin system inhibitor; *UPE* urinary protein excretion

We observed a statistically significant difference in the urine protein change among the hydroxychloroquine, leflunomide and renin-angiotensin system inhibitor-only groups [70.36 (57.54, 79.33)% vs. 57.29 (46.79, 67.29)% vs. 41.20 (25.76, 48.94)%, *P* < 0.001, *P*_1,2_ < 0.001, *P*_1,3_ < 0.001, *P*_2,3_ < 0.001)] (Fig. 1).

### Hematuria

At the end of the 6-month follow-up, all three groups showed decreasing hematuria. Red blood cell counts in the hydroxychloroquine group decreased from 11.62 (4.09, 24.20) to 8.65 (4.11, 18.15) per high power field at 6 months, but this decrease was not statistically significant (*P* = 0.362). Similarly, red blood cell counts in the renin-angiotensin system inhibitor-only group decreased from 14.17 (3.40, 19.73) to 11.65 (3.59, 16.48) per high power field at 6 months, but without statistical significance (*P* = 0.487). However, red blood cell counts in the leflunomide group significantly decreased from 43.75 (31.33, 67.90) at baseline to 15.13 (9.91, 23.53) per high power field at 6 months (*P* < 0.001)(Table 3).

**Table 3:** Changes in hematuria (u-RBC/HPF)

	Month0	Month1	Month3	Month6	*P*-value
HCQ group	11.62 (4.09, 24.20)	11.20 (4.69, 19.84)	10.26 (4.29, 19.65)	8.65 (4.11, 18.15)	0.362
LEF group	43.75 (31.33, 67.90)	36.66 (24.00, 58.69)	23.45 (17.26, 38.34)	15.13 (9.91, 23.53)	< 0.001
RASI-only group	14.17 (3.40, 19.73)	12.01 (3.30, 20.13)	13.28 (3.59, 17.94)	11.65 (3.59, 16.48)	0.487
*P*-value	< 0.001	< 0.001	< 0.001	< 0.001	–

*HCQ* Hydroxychloroquine, *LEF* leflunomide*, RASI *renin-angiotensin system inhibitor, *RBC* red blood cell, *HPF* high power field

**Fig. 2:**
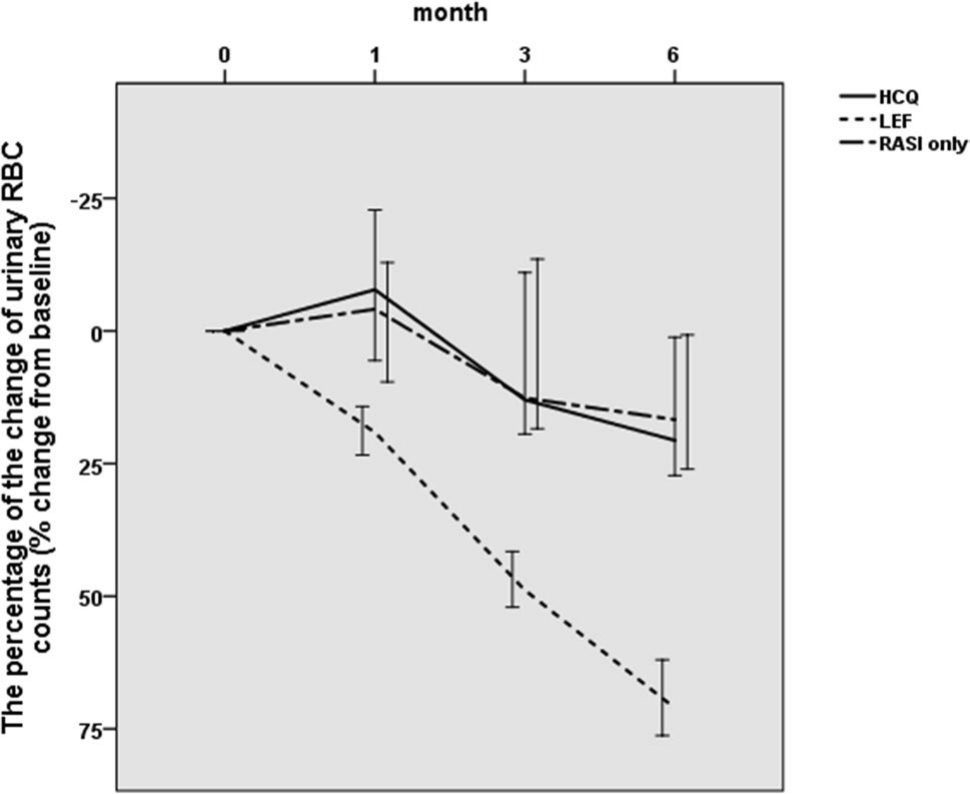
The percentage change in urinary RBC counts as a function of treatment (expressed as urinary red blood cell counts per high-power field). *HCQ* hydroxychloroquine; *LEF* leflunomide; *RASI* renin-angiotensin system inhibitor; *RBC* red blood cell

There was a statistically significant difference in the change in hematuria among the leflunomide, hydroxychloroquine and renin-angiotensin system inhibitor-only groups [20.62 (− 23.41, 33.34)% vs. 71.07 (56.48, 82.47)% vs. 16.71 (− 18.03, 28.11)%, *P* < 0.001, *P*_1,2_ < 0.001, *P*_1,3_ < 0.001, *P*_2,3_ = 0.717)] (Fig. 2), which showed that leflunomide had a significant advantage in improving hematuria in IgAN.

### eGFR

There was no significant difference in the baseline eGFR among the three groups (*P* = 0.128). After the end of the 6-month follow-up, the eGFR increased from 74.83 ± 14.14 to 77.39 ± 13.48 ml/min/1.73 m^2^ in the hydroxychloroquine group, from 79.86 ± 10.19 to 82.29 ± 9.83 ml/min/1.73 m^2^ in the leflunomide group, and from 79.26 ± 17.22 to 80.65 ± 17.03 ml/min/1.73 m^2^ in the renin-angiotensin system inhibitor-only group. Although all three groups showed increasing levels of eGFR, these changes were not statistically significant (*P* > 0.05) (Table 4).

**Table 4:** Estimated glomerular filtration rate during the 6-month follow-up (ml/min/1.73 m^2^)

	Month0	Month1	Month3	Month6	*P*-value
HCQ group	74.83 ± 14.14	75.50 ± 14.20	75.98 ± 13.69	77.39 ± 13.48	0.791
LEF group	79.86 ± 10.19	80.38 ± 10.19	81.16 ± 9.95	82.29 ± 9.83	0.632
RASI-only group	79.26 ± 17.22	79.03 ± 16.83	79.87 ± 16.77	80.65 ± 17.03	0.965
*P*-value	0.128	0.172	0.123	0.167	–

*HCQ* Hydroxychloroquine, *LEF* leflunomide*, RASI *renin-angiotensin system inhibitor

**Fig. 3:**
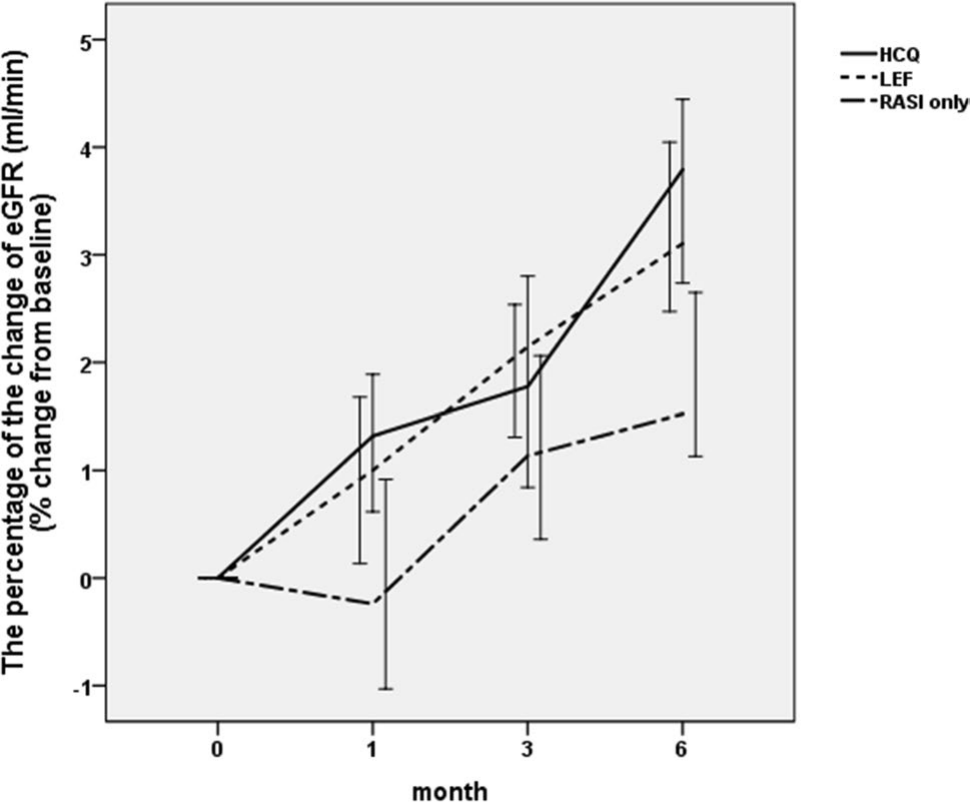
The percentage change in the eGFR as a function of treatment. *HCQ* Hydroxychloroquine; *LEF* leflunomide; *RASI* renin-angiotensin system inhibitor; *eGFR* estimated glomerular filtration rate

There was a statistically significant difference in the change in the eGFR among the hydroxychloroquine, leflunomide and renin-angiotensin system inhibitor-only groups (3.72 ± 2.97% vs. 3.16 ± 2.00% vs. 1.91 ± 2.41%, *P* = 0.001, *P*_1,2_ = 0.514, *P*_1,3_ = 0.001, *P*_2,3_ = 0.002) (Fig. 3), which suggested that additional use of hydroxychloroquine or leflunomide on top of a renin-angiotensin system inhibitor may be beneficial in delaying the progression of renal function.

### Adverse events

There were no serious side effects that caused a change in treatment regimen in any of the three groups. Although the levels of alanine aminotransferase were not significantly different among the three groups during the follow-up period, five patients in the leflunomide group and three patients in the hydroxychloroquine group had elevated levels of alanine aminotransferase that recovered after hepatoprotective treatment. Two patients in the leflunomide group had alopecia, albeit not requiring leflunomide discontinuation.

## Discussion

This study retrospectively explored the efficacy and safety of hydroxychloroquine and leflunomide in patients with IgAN with moderate proteinuria and mild to moderate renal function impairment (eGFR > 50 ml/min), and showed that both hydroxychloroquine and leflunomide were effective in reducing proteinuria. The advantage of hydroxychloroquine was a more significant reduction in proteinuria. The advantage of leflunomide was a more significant improvement in hematuria. No serious adverse events occurred in any of the three groups during the follow-up period. Therefore, our data suggest that hydroxychloroquine and leflunomide can be safely and effectively used to treat IgAN.

In this study, hydroxychloroquine showed a greater advantage in reducing proteinuria than leflunomide. Previous studies have shown that hydroxychloroquine has immunomodulatory effects by blocking Toll-like receptors 3, 7, 8, and 9 [25] and inhibiting the production of IL-6, IFN-α, and TNF-α [26], which may ameliorate the class switch recombination of IgA and glomerular mesangial matrix accumulation and glomerulosclerosis in patients with IgA nephropathy [27–29]. Another recent study showed that hydroxychloroquine inhibits the activation of the NF-κB/NLRP3 inflammasome pathway, thereby improving renal function in a rat model of IgAN [30].

Hematuria is the most typical presentation of IgAN, and microscopic hematuria is present in 70–100% of cases [31]. Nevertheless, hematuria has been ignored by researchers for a long time, and there are even studies supporting that hematuria is a benign feature of IgAN [32]. In the recent past, an observational study with a follow-up period of more than 20 years and a population of 1 million people showed that persistent isolated microscopic hematuria significantly increased the risk of end-stage renal disease [4], and some recent studies further highlighted that the levels of hematuria were independently associated with kidney disease progression, whereas hematuria remission was associated with improved kidney outcomes in IgAN patients with persistent proteinuria [33–35]. Our data showed that leflunomide improved hematuria significantly in patients with IgAN, which suggests that leflunomide could be recommended for IgAN patients with predominant hematuria.

The immunosuppressive mechanisms of leflunomide in IgAN remain unclear. An inhibitor of dihydrogenate dehydrogenase, leflunomide blocks the de novo pathway of pyrimidine nucleotide synthesis, thereby preventing the proliferation of T and B lymphocytes and immune responses, and inhibits the activity of tyrosine kinases and NF-κB in T lymphocytes [36, 37]. However, the mechanisms of action of leflunomide in improving hematuria need to be explored in further studies.

This study has limitations. First, it was a single-center retrospective study. There were only 159 enrolled patients, and the study time was only 6 months. The small scale of this study may lead to some variability and bias, so the conclusions need to be validated in further large-scale clinical trials. Second, according to our previous clinical experience, we preferred administering leflunomide in IgAN patients with severe hematuria, which caused an imbalance in the baseline data of hematuria among the three groups. Nevertheless, we analyzed all the eligible patients according to the inclusion criteria to reduce selection bias. To decrease baseline imbalance, we compared the change in urinary red blood cell counts instead of the specific levels at the selected time points. In addition, the mechanisms and targets of hydroxychloroquine and leflunomide in IgA nephropathy were not explored in this study, and it is hoped that future research will provide a comprehensive explanation.

## Conclusions

Our study retrospectively analyzed the efficacy and safety of hydroxychloroquine and leflunomide in patients with IgAN with moderate proteinuria and mild to moderate renal insufficiency, and the results showed that both hydroxychloroquine combined with a renin-angiotensin system inhibitor, and leflunomide combined with a renin-angiotensin system inhibitor were more effective than a renin-angiotensin system inhibitor alone in improving proteinuria and stabilizing renal function in IgAN patients. Leflunomide significantly improved hematuria. Hydroxychloroquine was more effective in reducing proteinuria, while leflunomide was superior in reducing hematuria. Neither drug significantly increased the occurrence of adverse events. The results need to be validated by future large-scale randomized controlled trials.

## Data Availability

The data for this study can be obtained from the corresponding author upon reasonable request.
